# Neglected Tropical Diseases outside the Tropics

**DOI:** 10.1371/journal.pntd.0000762

**Published:** 2010-07-27

**Authors:** Francesca F. Norman, Ana Pérez de Ayala, José-Antonio Pérez-Molina, Begoña Monge-Maillo, Pilar Zamarrón, Rogelio López-Vélez

**Affiliations:** Tropical Medicine Unit, Infectious Diseases Department, Hospital Ramón y Cajal, Madrid, Spain; University of Maryland School of Medicine, United States of America

## Abstract

**Background:**

The neglected tropical diseases (NTDs) cause significant morbidity and mortality worldwide. Due to the growth in international travel and immigration, NTDs may be diagnosed in countries of the western world, but there has been no specific focus in the literature on imported NTDs.

**Methods:**

Retrospective study of a cohort of immigrants and travelers diagnosed with one of the 13 core NTDs at a Tropical Medicine Referral Unit in Spain during the period April 1989-December 2007. Area of origin or travel was recorded and analyzed.

**Results:**

There were 6168 patients (2634 immigrants, 3277 travelers and 257 VFR travelers) in the cohort. NTDs occurred more frequently in immigrants, followed by VFR travelers and then by other travelers (p<0.001 for trend). The main NTDs diagnosed in immigrants were onchocerciasis (n = 240, 9.1%) acquired mainly in sub-Saharan Africa, Chagas disease (n = 95, 3.6%) in immigrants from South America, and ascariasis (n = 86, 3.3%) found mainly in immigrants from sub-Saharan Africa. Most frequent NTDs in travelers were: schistosomiasis (n = 43, 1.3%), onchocerciasis (n = 17, 0.5%) and ascariasis (n = 16, 0.5%), and all were mainly acquired in sub-Saharan Africa. The main NTDs diagnosed in VFR travelers were onchocerciasis (n = 14, 5.4%), and schistosomiasis (n = 2, 0.8%).

**Conclusions:**

The concept of imported NTDs is emerging as these infections acquire a more public profile. Specific issues such as the possibility of non-vectorial transmission outside endemic areas and how some eradication programmes in endemic countries may have an impact even in non-tropical western countries are addressed. Recognising NTDs even outside tropical settings would allow specific prevention and control measures to be implemented and may create unique opportunities for research in future.

## Author Summary

Neglected Tropical Diseases (NTDs) have been targeted due to their prevalence and the burden of disease they cause globally, but there has been no significant focus in the literature on the subject of NTDs as a group in immigrants and travelers, and no specific studies on the emerging phenomenon of imported NTDs. We present the experience of a Tropical Medicine Unit in a major European city, over a 19-year period, describing and comparing NTDs diagnosed amongst immigrants, travelers and travelers visiting friends and relatives (VFRs). NTDs were diagnosed outside tropical areas and occurred more frequently in immigrants, followed by VFR travelers and then by other travelers. The main NTDs diagnosed in immigrants were onchocerciasis, Chagas disease and ascariasis; most frequent NTDs in travelers were schistosomiasis, onchocerciasis and ascariasis, and onchocerciasis and schistosomiasis in VFRs. Issues focusing on modes of transmission outside endemic areas and how eradication programs for some NTDs in endemic countries may have an impact in non-tropical Western countries by decreasing disease burden in immigrants, are addressed. Adherence to basic precautions such as safe consumption of food/water and protection against arthropod bites could help prevent many NTDs in travelers.

## Introduction

The neglected tropical diseases (NTDs) are a group of mainly chronic illnesses identified as causing considerable morbidity and mortality typically amongst the world's poorest populations and which have also been shown to promote poverty [1]. The effect of the NTDs as a group is such that they have been considered to be part of a group of four, together with HIV/AIDS, tuberculosis (TB) and malaria. NTDs cause over 500, 000 deaths annually and have been estimated to result in a greater number of disability-adjusted life years lost than malaria and tuberculosis [2], [3].

Although in this sense many infections could be included, 13 core NTDs have been specifically targeted due to their prevalence and the burden of disease they cause globally and they include helminths, protozoa and tropical bacteria [1], [2].

Experts have argued other infections, such as those caused by arboviruses like dengue and yellow fever [4], and those caused by helminths like *Strongyloides* sp [5], should also be included and this will occur as efforts to control these diseases as a means to target poverty continue to develop. In the year 2000, the Millennium Declaration established goals to eliminate extreme poverty, hunger and disease and specifically addressed the health and economic impact of infectious diseases [1]. However, the NTDs remain relatively unknown to the general public, partly as a consequence of the decreased media coverage of these infections [6]. There has also been decreased investment by the pharmaceutical companies in new treatments for these conditions [7]. Recent increased efforts and interest at the international level have focused on the diseases mainly in endemic areas.

The growth in international travel and immigration which is taking place currently is a well-known phenomenon, and NTDs are no longer geographically-restricted as both immigrants and travelers (including immigrants who travel to visit friends and relatives, VFRs) in the western world may present with these infections. This study focuses on imported NTDs from the perspective of a Tropical Medicine Referral Unit in a European country which, with the exception of leishmaniasis, is not endemic for these infections. To date, there has been no significant focus in the literature on the subject of NTDs as a group in immigrants and travelers, and no specific studies on the emerging phenomenon of imported NTDs.

## Methods

### Ethics statement

This was a retrospective analysis of data obtained over a 19-year period, data were analyzed anonymously and written informed consent was not obtained from individual participants. The research was approved by the Ramón y Cajal Hospital's Ethics Committee (Comité Etico de Investigación Clínica, CEIC, Hospital Ramón y Cajal).

A retrospective study of a cohort of 6168 patients (2634 immigrants, 3277 travelers and 257 VFRs- travelers visiting friends and relatives) seen at a Tropical Medicine Referral Unit in Madrid, Spain during the period April 1989 to December 2007 was performed. The unit is part of the Infectious Diseases department of a large (>1200-bed) tertiary referral teaching hospital and sees a large proportion of sub-Saharan immigrants referred from a non-profit organization for African immigrants based in Madrid as well as a large subset of Latin American immigrants. In Spain, basic medical assistance is given to all the population, national or foreign, and to both legal and illegal immigrants. Patients need only to be in possession of a health card to receive medical care. If there are difficulties in accessing this health card (cultural/linguistic barriers), patients may be referred from non-profit organizations.

A traveler was defined as any person who had crossed an international border within 10 years before presentation and an immigrant was defined as any person arriving in a country different from their own with the objective of settling in the new country. A VFR traveler was defined as any traveler (or their children, even if they were born in Spain) who returned to their country of origin for the purpose of visiting friends and relatives.

Screening for asymptomatic patients comprised blood count, biochemistry, basic urinalysis, HIV, hepatitis B virus and hepatitis C virus serology, rapid plasma reagin for syphilis, tuberculin skin test, stool parasites, PCR for malaria in sub-Saharan Africans (since 2005), and *Trypanosoma cruzi* serology (both immunofluorescent antibody test and ELISA) in Latin Americans. Other tests were ordered according to specific symptoms and signs.

Cases were identified from a database of patients if they had one or more of the 13 core NTDs (ascariasis, trichuriasis, hookworm infection, schistosomiasis, lymphatic filariasis, trachoma, onchocerciasis, leishmaniasis, Chagas disease, leprosy, human African trypanosomiasis, dracunculiasis, Buruli ulcer) [1]. Gender, age and country of origin/travel were recorded. Standard diagnostic methods for each infection were used. A patient was defined as having NTD polyparasitism if more than one NTD was diagnosed. Positive epidemiological risk factors were considered in immigrants from endemic areas for the infection and if a traveler had visited an endemic area and had history of exposure.

Cases of schistosomiasis, onchocerciasis and lymphatic filariasis were classified as definite or probable cases based on the evidence available as outlined below.

### Ascariasis, trichuriasis, hookworm

Diagnoses based on finding typical parasite eggs on stool examination (formol ether concentration method).

### Schistosomiasis

Definite schistosomiasis in an immigrant was diagnosed after identifying the parasite eggs in specimens (urine, stool or biopsy specimens). Probable schistosomiasis in an immigrant was diagnosed on finding positive serology in a patient with clinical symptoms/signs of schistosomiasis (hematuria and/or eosinophilia) or if there was documented response to treatment with praziquantel (resolution of symptoms/signs and normal eosinophil count) in a patient with clinical symptoms/signs of schistosomiasis (hematuria and/or eosinophilia). Definite schistosomiasis in a traveler was defined after identifying the parasite eggs in specimens (urine, stool or biopsy specimens) or if history of exposure and positive serology. Probable schistosomiasis in a traveler was diagnosed if history of exposure and suggestive clinical signs/symptoms (swimmer's itch, Katayama fever or hematuria and/or eosinophilia) or history of exposure, suggestive clinical symptoms/signs and response to praziquantel (resolution of signs/symptoms and normal eosinophil count).

### Lymphatic filariasis

Diagnosis established on finding microfilariae after lysis-centrifugation of night-time blood specimens. Probable cases of lymphatic filariasis based on epidemiological risk factors, compatible clinical symptoms/signs and response to treatment.

### Onchocerciasis

Diagnosis of definite onchocerciasis in immigrants was based on identification of *Onchocerca volvulus* microfilariae in “skin snips” or clinical signs/symptoms of onchocerciasis (pruritus and/or skin lesions suggestive of onchocerciasis) and a positive Mazzotti provocation test with diethyl carbamazine (DEC) (symptoms appearing after a single oral dose of 25–50 mg of DEC, performed in patients with negative skin snips and no evidence of ocular involvement). Probable onchocerciasis in an immigrant was diagnosed if clinical signs/symptoms and response to treatment with ivermectin (symptom resolution). Onchocerciasis (definite) was diagnosed in a traveler when microfilariae were identified in skin snips or if positive serology in a patient with clinical symptoms/signs of onchocerciasis (pruritus/skin lesions suggestive of onchocerciasis). Slit lamp examinations were performed in patients with ocular symptoms or prior to the Mazzotti provocation test.

### Leishmaniasis

Diagnosis based on identification of amastigotes in skin or bone marrow biopsies, and/or positive culture results (Novy-McNeal-Nicolle medium) and/or positive PCR results (*Leishmania* nested PCR, LnPCR, of blood/bone marrow/skin biopsies)[8].

### Chagas disease

*T. cruzi* infection was defined by positive serology using two different techniques (ELISA and IFAT). For all patients with Chagas disease, an ECG and echocardiogram were requested, and upper/lower gastrointestinal barium studies or esophageal manometry were requested in patients with symptoms.

### Leprosy

Cases were diagnosed on obtaining positive skin smears (presence of Ziehl-Nielsen acid fast bacilli) in a patient with clinical symptoms/signs of leprosy.

An SPSS 12.0 for Windows package (SPSS Inc., Chicago, USA) was used to analyze the data.

## Results

Out of 6168 patients, 2634 were immigrants, 3277 travelers and 257 were VFR travelers. In the group of immigrants there were 1567 males (59.5%) and 1067 females (40.5%). The mean age was 30 years (range: 1–83 yrs). The majority of immigrants were from Central Africa (n = 912, 34.6%), West Africa (n = 843, 32.0%), and South America (n = 603, 22.9%). In the group of travelers there were 1704 males (52.0%) and 1573 females (48.0%). The mean age was 36 years (range: 21–80 yrs). The majority had traveled to Central America and the Caribbean (n = 620, 18.9%), West Africa (n = 509, 15.5%), and South America (n = 508, 15.5%). In the group of VFR travelers there were 123 males (47.9%) and 134 females (52.1%). The mean age was 36 years (range 1–73 yrs). The majority had traveled to Central Africa (n = 109, 42.4%), South America (n = 60, 23.3%) and West Africa (n = 50, 19.5%). Patient distribution according to world area of origin (for immigrants) or world area of travel (for travelers and VFR travelers) is shown in table 1. The number of NTDs diagnosed in immigrants, travelers and VFR travelers during the study period is shown in table 2. NTDs occurred more frequently in immigrants, followed by VFR travelers and then by other travelers. This difference was found to be statistically significant (chi-square test, p<0.001 for trend).

**Table 1:** Age, gender and patient distribution according to world area of origin or travel.

	Immigrants n = 2634(% of total)	Travelers n = 3277(% of total)	VFR n = 257(% of total)
Mean age (years)	30	36	36
Gender (% male)	59.5%	52.0%	47.9%
World area of origin or travel:			
Central Africa	912 (34.6%)	455 (13.9%)	109 (42.4%)
East Africa	52 (2.0%)	478 (14.6%)	5 (1.9%)
North Africa	53 (2.0%)	110 (3.4%)	3 (1.2%)
West Africa	843 (32.0%)	509 (15.5%)	50 (19.5%)
South Africa	3 (0.1%)	69 (2.1%)	0 (0%)
Central America/Caribbean	81 (3.1%)	620 (19.0%)	20 (7.8%)
North America	1 (<0.1%)	5 (0.2%)	1 (0.4%)
South America	603 (22.9%)	508 (15.5%)	60 (23.3%)
East Asia	9 (0.3%)	17 (0.5%)	0 (0%)
West and Southwest Asia	9 (0.3%)	33 (1.0%)	1 (0.4%)
Southcentral Asia	26 (1.0%)	287 (8.8%)	5 (1.9%)
Southeast Asia	7 (0.3%)	162 (4.9%)	3 (1.2%)
Oceania	1 (<0.1%)	14 (0.4%)	0 (0%)
Europe	31 (1.2%)	7 (0.2%)	0 (0%)
Not recorded	3 (0.1%)	3 (0.1%)	0 (0%)

**Table 2:** NTDs diagnosed in immigrants, travelers and VFRs (April 1989- December 2007).

NTD*	Totaln = 6168	Immigrantsn = 2634(% of total)	Travelersn = 3277(% of total)	VFRn = 257(% of total)
Ascariasis	103	86 (3.3%)	16 (0.5%)	1 (0.4%)
Trichuriasis	74	74 (2.8%)	0 (0.0%)	0 (0%)
Hookworm infection	11	9 (0.3%)	2 (0.1%)	0 (0%)
Schistosomiasis	73	28 (1.1%)	43 (1.3%)	2 (0.8%)
Lymphatic filariasis	2	1 (<0.1%)	1 (<0.1%)	0 (0%)
Trachoma	0	0 (0.0%)	0 (0.0%)	0 (0%)
Onchocerciasis	271	240 (9.1%)	17 (0.5%)	14 (5.4%)
Leishmaniasis (cutaneous-mucocutaneous/visceral)	13/2	5/1 (0.2%)	8/1 (0.3%)	0 (0%)
Chagas disease	95	95 (3.6%)	0 (0.0%)	0 (0%)
Leprosy	10	9 (0.3%)	1 (<0.1%)	0 (0%)
Human African trypanosomiasis	0	0 (0.0%)	0 (0.0%)	0 (0%)
Dracunculiasis	0	0 (0.0%)	0 (0.0%)	0 (0%)
Buruli ulcer	0	0 (0.0%)	0 (0.0%)	0 (0%)
Total number of patients diagnosed with ≥1 NTD**	576 (9.3%)	476 (18.1%)	85 (2.6%)	15 (5.8%)
Total number of patients with >1 NTD (polyparasitised)	78 (1.3%)	72 (2.7%)	4 (0.1%)	2 (0.8%)

*Core neglected tropical diseases ranked by global prevalence (*1*).

**As each patient could have >1 diagnosis, the number of cases may be higher than the number of patients.

### Soil-transmitted helminths

In total, 103 patients were diagnosed with ascariasis (86 immigrants, 16 travelers, and 1 VFR). Amongst the immigrants there were 54 females and 32 males (mean age 24 years, range: 1–74 yrs). Most patients were from Central Africa [Equatorial Guinea (68), Angola (3), Cameroon (1), and Central African Republic (1)], followed by South America [Ecuador (6), Colombia (3)], and West Africa [Liberia (2), Nigeria (2)]. There were eight male and eight female travelers (mean age 32 years, range 23–80 yrs). The majority had traveled to sub-Saharan Africa: Equatorial Guinea (3), and one each to Congo D.R., Mozambique, Senegal, and Cameroon. Other countries visited included: India (2), Ecuador (2), Morocco (2), Algeria, Mexico, and Guatemala. One female VFR traveler to E. Guinea was diagnosed with ascariasis.

All the patients diagnosed with trichuriasis were immigrants (47 females and 27 males, mean age 26 years, range: 1–73 yrs), the majority from Central Africa [Equatorial Guinea (65), Angola (2), Cameroon (1)] followed by Ecuador (2) and one each from Brazil, Dominican Republic, Guatemala, and Liberia.

Out of 11 hookworm infections, nine occurred in immigrants (five females, four males, mean age: 35 years, range: 9–69 yrs) and two in travelers (both male, both aged 29 yrs). All the immigrants were originally from Equatorial Guinea, and the travelers had visited Senegal/Guinea Bissau and Thailand.

### Schistosomiasis

There were 28 immigrants diagnosed with schistosomiasis (13 definite and 15 probable cases), 26 males and two females (mean age 25 years, range: 16–55 yrs). Out of the 13 definite cases, 8 had *S. haematobium* and 5 had *S. mansoni*. The majority (26) of patients were from sub-Saharan Africa. Patient distribution according to country of origin was as follows: Equatorial Guinea (5), Mali (5), Cameroon (3), Nigeria (3), Sierra Leone (2), Angola (2), Ghana (2), Liberia (2), Niger (1), Republic of Guinea (1), Egypt (1), and Ecuador (1). With respect to the main symptoms/signs: no patients presented with acute symptoms, 18 (64.3%) had eosinophilia, and 13 (46.4%) had hematuria.

Out of 43 cases of schistosomiasis diagnosed in travelers, 19 were considered definite and 24 were probable cases, 23 were male and 20 female (mean age 34 years, range 23–55 yrs). Out of 19 definite cases, 5 had *S. haematobium* and 14 were classified as *Schistosoma* sp. (positive serology and history of exposure). For all travelers, risk factors for acquisition of schistosomiasis were identified (freshwater exposure in endemic countries), and a large proportion of patients (19/43, 44.2%) had traveled to Mali. With respect to main symptoms/signs: 7 patients (16.3%) referred symptoms suggestive of acute schistosomiasis, 32 patients (74.4%) had eosinophilia, and 17 (39.5%) had hematuria.

Only two cases were diagnosed amongst VFRs: one 53 year-old female (*Schistosoma* sp., traveled to E. Guinea) and one35 year-old male (*S. haematobium*, traveled to Cameroon) were diagnosed with schistosomiasis.

### Lymphatic filariasis

Two probable cases of lymphatic filariasis were identified (microfilariae were not obtained from specimens but they had suggestive clinical signs/symptoms and responded to treatment). A 60 year-old male immigrant from India presented with eosinophilia, bilateral testicular hydrocele and prominent chyluria. A 52 year-old Spanish male who had worked in Equatorial Guinea for 2.5 years had presented with eosinophilia, bilateral lower limb edema and a left testicular hydrocele.

### Onchocerciasis

A total of 240 cases of onchocerciasis were identified in immigrants. Of these, 169 cases were definite cases and 71 were probable cases. There were 158 females and 82 males (mean age 33 years, range: 5–76 yrs). All but two of the cases of onchocerciasis in immigrants occurred in African patients. The majority of patients were from Equatorial Guinea (213/240, 88.8%), followed by Cameroon (6/240, 2.5%), Nigeria (4/240, 1.7%), Angola (4/240, 1.7%), Zaire (3/240, 1.3%) and one each from Republic of Guinea, Mali, Togo, D.R. Congo, Ghana, Sierra Leone, Sao Tome, Ivory Coast, Colombia, and Ecuador (1/240, 0.4%).

**Figure 1:**
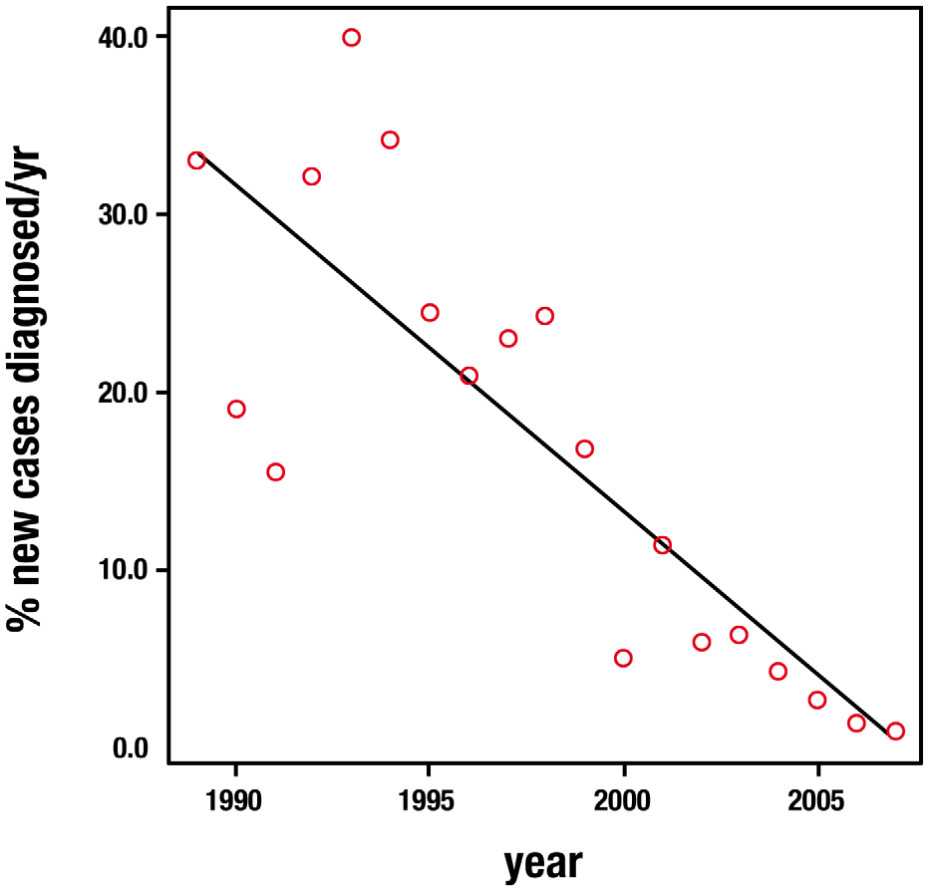
Linear regression analysis: number of new cases of onchocerciasis per new African patients seen at the unit each year. p<0.001; β −1.84 (95% C.I. −2.46 to −1.22).

A linear regression analysis was performed taking into account the number of new diagnoses of onchocerciasis (excluding two patients from South America) and the number of new African immigrants seen per year. A statistically significant decrease was found in the number of cases of onchocerciasis diagnosed each year at the unit (p<0.001; β −1.84, 95% C.I. −2.46 to −1.22) (See figure 1).

There were 17 travelers (nine females and eight males, mean age 37 years, range: 21–59 yrs) diagnosed with definite onchocerciasis. Of these, 16 patients were long-term travelers, (trip duration >3 months, range: 3–336 months) and 1 patient had traveled for 1 month. All patients had traveled to sub-Saharan Africa and countries visited included: Equatorial Guinea, Angola, Cameroon, DR of Congo, Ivory Coast, Burkina Faso, Central African Republic and Mali. Some patients had visited more than one country during their travel.

Amongst VFR travelers, 14 diagnoses of onchocerciasis were made (5 males and 9 females, mean age 31 years, range: 6–53 yrs), 12 had traveled to E. Guinea and 2 had traveled to Cameroon.

### Leishmaniasis

Amongst the group of immigrants, there were three males and three females (mean age: 34 years, range: 17–50 yrs). There were three cases of cutaneous leishmaniasis (CL) (patients were from Brazil, Algeria and Panama), two cases of mucocutaneous leishmaniasis (ML) (from Bolivia and Ecuador) and one visceral leishmaniasis (VL) (from Cameroon but had stayed in Northern Africa for two years).

There were seven male and two female travelers (mean age: 37 years, range 28–55 years). CL was diagnosed in six (these travelers had visited Costa Rica (n = 2), Panama, French Guyana, Colombia and India), ML in two (from Ecuador and Bolivia/Peru) and VL in one patient (Peru).

### Chagas disease

In total, 95 immigrants were diagnosed with Chagas disease: 62 females and 33 males, (mean age 36 years, range: 16–69 years). The majority of patients were from Bolivia (90/95, 94.7%) and one each from Brazil, Chile, Ecuador, Paraguay, and Honduras. With regards to risk factors for acquisition of Chagas disease in their countries of origin, 79 patients were from rural areas, 76 patients recalled having seen the vector in their homes in their countries of origin, 15 patients had received a blood transfusion in endemic countries and for 7 patients vertical transmission was a possibility (mother with known positive *T. cruzi* serology). Not all patients had completed their full work-up to ascertain degree of visceral involvement during the study period: 16 patients were found to have abnormalities on ECG (out of 68 tests performed) and 10 had abnormalities on echocardiogram suggestive of Chagas disease (out of 58 tests performed). Out of 36 esophageal manometries and 44 barium enemas performed 4 in each group, showed abnormalities suggestive of Chagas disease.

### Leprosy

Nine immigrants were diagnosed with leprosy, five males and four females (mean age 42 years, range: 24–64 yrs). Two patients were from the Dominican Republic, two from Colombia, two from Equatorial Guinea and one each from Mauritania, Brazil, and the Philippines. Four patients had lepromatous leprosy, two patients, tuberculoid leprosy, one patient, borderline-tuberculoid leprosy, one had borderline-lepromatous leprosy and one had borderline leprosy. The one traveler diagnosed with leprosy had lived for nearly 60 years in Cuba (expatriate) and was diagnosed with borderline-lepromatous leprosy.

There were no cases of trichuriasis, hookworm infection, lymphatic filariasis, leishmaniasis, Chagas disease, or leprosy diagnosed in VFR travelers.

### Trachoma, human African trypanosomiasis, dracunculiasis, Buruli ulcer

No cases were diagnosed in this series.

### NTD polyparasitism

Some patients were diagnosed with more than one NTD (with a maximum of 2 NTDs per patient): 72 immigrants, 4 travelers and 2 VFR travelers.

In the group of immigrants, 2 were diagnosed with hookworm infection and ascariasis (both from E. Guinea), 3 were diagnosed with hookworm and trichuriasis (all from E. Guinea), 8 were diagnosed with ascariasis and onchocerciasis (all from E. Guinea), 49 were diagnosed with ascariasis and trichuriasis (44 from E. Guinea, 2 from Ecuador and one each from Liberia, Angola and Cameroon), 1 patient was diagnosed with CL and trichuriasis (from Brazil), 2 with leprosy and onchocerciasis (from E. Guinea) and 7 with onchocerciasis and trichuriasis (all from E. Guinea).

In the group of travelers, 1 patient was diagnosed with hookworm and ascariasis (travel to Brazil for 25 days), 1 patient with ascariasis and onchocerciasis (travel to Cameroon for 6 months), and 2 patients were diagnosed with onchocerciasis and schistosomiasis (travel to Central African Republic for 5months and the other patient had traveled to Burkina Faso and Ivory Coast for 1 month).

In the group of VFR travelers, 2 patients were diagnosed with schistosomiasis and onchocerciasis. These patients had traveled to Cameroon and Equatorial Guinea for 15 days and 2 months, respectively.

NTD polyparasitism was most frequent amongst immigrants, followed by VFR travelers and then by other travelers, respectively (p<0.001 for trend).

## Discussion

NTDs are acquiring a more public profile, and in parallel the concept of imported NTDs, is also emerging. As demonstrated, NTDs are not restricted to the tropics, and may be diagnosed in countries of the developed world in travelers, immigrants and VFRs.

Certain limitations of the study, such as the under-representation of patients from Asia, Oceania and North America, may not allow extrapolation of all results to other groups of travelers and immigrants.

Some patients were lost to follow-up reflecting the mobile nature and difficulty of access to health services of both immigrants (illegal residents were also included in the study) and travelers. This may have led to an underestimation of the frequency of NTDs in this group, especially if loss of follow-up implied a diagnosis was not reached. However, the large sample size and the length of the study period add strength to the study, and highlight some important issues.

NTDs manifesting in endemic areas and imported NTDs will share certain features but differences may also be expected. The spectrum and frequency of diseases diagnosed in endemic and non-endemic areas may differ. Disease burden may be lower when the infections occur outside endemic areas. Although the number of patients traveling with pre-existing medical conditions is increasing [9], a large proportion of travelers are healthy and in the same way an important proportion of immigrants who seek work opportunities and who are able to travel to do so, would generally be expected to be in good health. The occurrence of an NTD in a healthy person should not have the same devastating effects as those observed in endemic areas, where infections and re-infection with multiple NTDs are not infrequent and worsen the burden of disease.

Populations in endemic regions can be infected with multiple neglected tropical diseases [10] and this was also observed with imported NTDs. As would be expected due to the greater risk and length of exposure, NTD polyparasitism was most frequent amongst immigrants, followed by VFR travelers and other travelers, respectively, and this difference was statistically significant for trend. The most frequent cause of polyparasitism in this series was due to coinfection with one of the geohelminths (ascariasis, trichuriasis, and hookworm), reflecting the large burden of disease caused by these parasites worldwide [1].

There were few cases of NTDs amongst VFR travelers, who as a group accounted for only a small proportion of the study population (approximately 4%). Risk factors for acquisition of some of the NTDs (often chronic diseases) in VFRs may be due to exposure during more recent travel or may be related to exposure years previously before migration took place. Acquisition of other NTDs (such as *T. cruzi* infection which is very rarely diagnosed in travelers) is more likely to have occurred before migration than during VFR travel. VFRs have been shown to have an increased risk of acquiring infections during travel [11], and in this study NTDs were found to be significantly more frequent in VFR travelers compared to other travelers (but less frequent than in immigrants). However, further research on NTDs in VFR travelers would be of value and comparisons should be interpreted with caution.

An important difference was the large number of immigrants infected with *O. volvulus*, which was diagnosed infrequently in travelers. Most of the patients diagnosed with onchocerciasis were from Equatorial Guinea, which achieved independence from Spain in the 1960s. These historical links between both countries explain the large number of patients from E. Guinea, an area highly endemic for onchocerciasis, and therefore the high number of *O. volvulus* infections. Differences may arise due to the length/type of exposure necessary for acquisition of some infections, as these conditions are achieved more readily by the local population in endemic areas. However, with the recent changes in travel patterns, not only quantity but also quality of travel becomes an issue, as more people visit remote and exotic destinations. As occurred for some cases in this study, several case reports describe only short durations of exposure for filarial-infected travelers and risks may be linked to the lack of preventive measures and specific exposure in vector habitats [12], [13]. In future, more travelers may present to health centers in areas of the western world with filariasis.

On the other hand, most cases of schistosomiasis were diagnosed in travelers and this may be partly due to the presence of semi-immunity in adult immigrants from endemic areas. In communities where *Schistosoma* species are endemic, prevalence and intensity of schistosomiasis have been found to be higher in children whereas some adults may generate certain protective responses [14], [15]. These protective mechanisms following exposure would not be expected to have developed in travelers, especially following only short-term exposure.

Other NTDs, such as dracunculiasis, Buruli ulcer and African and American trypanosomiasis are very infrequent in travelers, with only very few cases reported in the literature [16]–[19], and these diseases were not diagnosed in this cohort either.

The absence of NTDs such as dracunculiasis even in the cohort of immigrants may reflect the decrease in incidence world wide which may be attributed to the progress of the global dracunculiasis eradication program [20]. The Global Program to Eliminate Lymphatic Filariasis has also had an important impact in communities in Africa and Asia [21]. An example of the success of another of these programs could be illustrated by the findings in this study, which showed a significant decrease in cases of onchocerciasis in recent years, despite the increasing number of patients attended at the unit. The Onchocerciasis Control Program in West Africa focusing on vector control and the Ivermectin donation program established in 1987, have increased general interest in health-related public-private partnerships [22], [23]. The results presented in this analysis may reflect how the implementation of such programs in an endemic developing country may have more widespread effects by changing the presentation and epidemiology of the disease in a non-endemic country.

Once NTDs are imported into non-endemic areas, the possibility of transmission and the resulting impact should be considered. In general, the epidemiology of infectious diseases will be influenced by the interactions between pathogen, host (human, animal or vector) and the environment [24], and most of these infections will have only limited transmission as the required vector may be absent and the environmental conditions unfavorable. However, there have been rising concerns regarding the emergence of some pathogens due to infectious agents being imported into novel non-endemic areas and the possibility of accidental spread of disease vectors between areas which may act as drivers for the emergence of infections [24]. Local vectors may become infected with imported infectious agents resulting in local cases as occurred in the first outbreak of chikungunya virus infection in a temperate country which was registered in Italy, where the vector, *Aedes albopictus* is already established [25]. Similar outbreaks could theoretically occur in other non-tropical countries and involving other vectors and other imported infectious pathogens. The prospect of possible future spread of some of the NTDs outside their usual geographical areas should therefore not be dismissed. Global changes in climate and temperatures may affect the distribution of vectors and trigger disease outbreaks and the possibility of non-vectorial transmission also emerges [24], [26].

Chagas disease, paradoxically, is an NTD with a reported decreasing health and economic impact in endemic countries due to the success of multi-national control programs aimed principally at the interruption of vectorial and transfusional transmission [27], but the disease now appears to be emerging outside these areas [28]. In Europe, and especially in Spain, cases of Chagas disease have been increasing due to the recent increase in immigration from Latin America and the disease may become an important cause of cardiomyopathy in the near future [28]. Tainted donor blood or organ grafts and vertical transmission would be the main modes of transmission of *Trypanosoma cruzi* imported by immigrants in countries where vectorial transmission does not occur. This growth in immigration has had sufficient impact to warrant changes in national legislation with respect to the screening of blood donations [29], and yet pregnant Latin American women are not always screened for *Trypanosoma cruzi* infection. Specific preventive strategies would need to be developed and implemented at a national level in order to control non-vectorial transmission outside endemic areas.

Health professionals attending immigrants and travelers from tropical countries should consider protocols for screening and prevention of NTDs in their everyday practice. Regarding pre-travel advice, for travelers and especially VFRs, adherence to a few basic precautions, (safe consumption of food/water, protection against arthropod bites and avoiding swimming in fresh water/walking barefoot) could help prevent the majority of NTDs. Early detection of cases may permit prevention of secondary transmission.

The control of NTDs in endemic areas with low-cost effective interventions such as rapid-impact packages, may lead to long-term economic growth given the reported high return rates on investments in these diseases [1], [21], [30], [31]. Recognising that certain NTDs may also have an impact in areas of the western world should help create unique opportunities for research and control measures in countries with greater means. Studies in non-endemic areas may provide valuable data as patients will not ordinarily be re-infected unless they travel to endemic countries again.

Hopefully the problem of the neglected tropical diseases “spilling over” into more developed countries will be linked to even greater combined international efforts to control these infections.

## Supporting Information

Checklist S1 STROBE Checklist.
(0.09 MB DOC)
